# Genetic Variants in CSMD1 Gene Are Associated with Cognitive Performance in Normal Elderly Population

**DOI:** 10.1155/2017/6293826

**Published:** 2017-12-12

**Authors:** Vadim Stepanov, Andrey Marusin, Kseniya Vagaitseva, Anna Bocharova, Oksana Makeeva

**Affiliations:** ^1^Institute of Medical Genetics, Tomsk National Medical Research Center, Tomsk, Russia; ^2^Tomsk State University, Tomsk, Russia; ^3^Nebbiolo Center for Clinical Trials, Tomsk, Russia

## Abstract

Recently, genetic markers rs10503253 and rs2616984 in the CUB and Sushi multiple domains-1 (CSMD1) gene have been reported to be associated with schizophrenia and cognitive functions in genome-wide association studies. We examined the associations of the above SNPs with cognitive performance evaluated by the Montreal Cognitive Assessment (MoCA) tool in a cohort of the normal elderly from the Russian population. Significant association of rs2616984 genotypes with the MoCA scores was found using nonparametric analysis. No association of rs10503253 with MoCA scores was observed using both parametric and nonparametric statistics. Significant combined effect of two-locus CSMD1 genotypes on MoCA scores was demonstrated by median test. Allele “A” and genotype “AA” of rs2616984 were significantly associated with the lower MoCA scores in comparison of 1st and 4th quartiles of MoCA total score distribution. The results suggest that genetic variants in CSMD1 gene are likely a part of genetic component of cognitive performance in the elderly.

## 1. Introduction

The single-nucleotide polymorphisms (SNPs) in the CUB and Sushi multiple domains-1 (CSMD1) gene were recently identified in genome-wide association studies (GWAS) as significant genetic markers for schizophrenia (SZ) and cognitive performance. CSMD1 gene spanning over 2.6 Mb on chromosome 8p23.2 is highly expressed in the central nervous system and epithelial tissues [1] and encodes an important cell adhesion molecule involved in the development, connection, and plasticity of brain circuits. Despite the fact that the exact role of CSMD1 in neurodevelopmental process is not clear, murine models indicate that CSMD1 knockout induces behaviors reminiscent of blunted emotional responses, anxiety, and depression, suggesting an influence of the CSMD1 on psychopathology and endophenotypes of the negative symptom spectra [2]. A common intronic CSMD1 variant, rs10503253, was reported as genome-wide significant for SZ by Schizophrenia Psychiatric Genome-Wide Association Study Consortium [3] and was subsequently replicated in other GWA and meta-analysis studies [4–6]. A minor allele “A” of rs10503253 is associated with deleterious effects across a number of neurocognitive phenotypes, such as poorer performance on neuropsychological measures of general cognitive ability and memory function in SZ patients [7], and affects general cognitive ability and executive function in healthy individuals [8]. Another intronic variant in CSMD1, rs2616984, located 302 kb away, was found as genome-wide significant for the performance on standardized cognitive tests [9]. Recently we have demonstrated the association of the latter genetic variant with both SZ and Alzheimer's disease in a Russian population [10, 11]. These data suggested that neuropsychological effects of CSMD1 and the plausible role of its genetic variation in schizophrenia and other neuropsychiatric diseases are based on common underlying neurological mechanism developed via cognitive endophenotypes. In this study, we investigated the role of common genetic variation in CSMD1 gene in cognitive performance in normal elderly population.

## 2. Material and Methods

708 elderly individuals of European Russian descent without dementia and neurological diseases were recruited from a population-based cohort study on primary prevention of Alzheimer's disease in Tomsk, Russia [12, 13]. The sample consisted of 74% of females (see Table 1). Mean age in the sample was 70.9 (varying from 60 to 89) years. Level of education of the study participants was assessed with two measures: number of years of education and achieved level of education. Level of education was relatively high with 42% of study participants having bachelor's or master's degree and 7% having doctoral degree. Only 7% of the sample had been exposed to memory testing previously with clinical or research purpose reasons.

Health characteristics are presented in Table 1. Health conditions were self-reported with the notice from investigator to report only on diagnoses established by medical doctors and confirmed in medical records. The sample had high prevalence of cardiovascular conditions with high blood pressure indicated in 81% of study subjects.

In each participant, the cognitive performance was assessed using the Montreal Cognitive Assessment (MoCA). MoCA measures 8 cognitive domains: memory, attention, naming, visuospatial/executive, language, abstraction, delayed recall, and orientation domains. MoCA scores ranged between 0 and 30 points, and higher scores indicate better cognitive function.

Genotyping of 2 genetic variants in CSMD1 gene, rs10503253 and rs2616984, was performed by multiplex PCR with the following iPLEX primer extension reaction and detection of allele-specific extension products by matrix-assisted laser desorption/ionization time-of-flight (MALDI-TOF) mass spectrometry on Sequenom MassARRAY 4 platform. Details of the genotyping method have been previously described elsewhere [14].

Linkage disequilibrium (LD) between genetic variants and haplotype frequencies were estimated using Haploview 4.2 software. Statistical analysis of relationships between genetic markers and cognitive performance was performed in Statistica 7.0 (StatSoft Inc.) package using parametric (analysis of variance, ANOVA) and nonparametric (Kruskal-Wallis test and median test) statistics. For ANOVA analysis, MoCA scores were adjusted for age and education using linear regression model. Differences in allele and genotype frequencies between quartiles of MoCA distribution were estimated by chi-square test.

## 3. Results

Allele and haplotype frequencies of rs1050325 and rs2616984 in the total sample of 708 elderly subjects, as well as in the first (MoCa < 21) and fourth (MoCA > 24) quartiles of MoCA scores distribution, are presented in Table 2. Genotype distribution for both genetic variants in CSMD1 gene corresponded to Hardy-Weinberg equilibrium. No difference in the allele frequency between men and women was found. Minor alleles of both SNPs demonstrated very similar frequency in the total sample: 0.267 for “A” allele of rs10503253 and 0.272 for “G” allele of rs2616984. Two intronic variants in CSMD1 gene, located 302 kb apart, show very low level of linkage disequilibrium (*D*′ = 0.107; LOD = 0.24).

Significantly higher frequency of the major allele of rs2616984 (“A”) was observed in a subsample of individuals with MoCA score less than 21 compared to fourth quartile's subsample (0.754 versus 0.674; chi-square = 6.008; *p* = 0.0142), while no significant differences between lower and upper quartiles were found for rs10503253.

Odds ratio values for lower MoCA score associated with allele “A” and genotype “AA” of rs2616984 were 1.49 (95% CI 1.07–2.07, *p* = 0.014) and 1.70 (95% CI 1.11–2.61, *p* = 0.009), respectively. Genotype “AG” of rs2616984 and haplotype “CG” of rs105032/rs2616984 were associated with higher MoCA score (Table 3).

One-way ANOVA demonstrates no significant differences in the mean MoCA scores among genotypes of both genetic variants in CSMD1 gene, as well as among combinations of genotypes of two SNPs (Table 4). However, the effect of rs2616984 on MoCA scores was close to statistically significant (*F* = 2.814, *p* = 0.060).

In addition, less conservative nonparametric statistics indicate that genetic variation in rs2616984 locus significantly influenced the MoCA values in the normal elderly population. Under codominant model, *p* value for median test was 0.024 and Kruskal-Wallis *p* was 0.020. No significant association of rs10503253 with MoCA scores was observed using both parametric and nonparametric statistics. Significant combined effect of two-locus CSMD1 genotypes on MoCA scores was demonstrated by median test (chi-square = 16.19; df = 8; *p* = 0.039).

## 4. Discussion

Current data on contribution of CSMD1 genetic variation to neuropsychiatric diseases and to cognitive performance are quite controversial. More evidence is accumulated on rs10503253, which is genome-wide significant marker for SZ in Europeans according to initial GWAS and subsequent replicative studies (see above), but is not replicated for Japanese [15] and Han Chinese [16]. Association of this genetic variant with cognitive and memory functions initially reported for European SZ patients [7] was confirmed for normal Greek population, but was not replicated in Norwegian healthy cohort [17]. Another intronic variant in CSMD1, rs2616984, was associated with cognitive performance in European GWAS [9] and recently was found as susceptibility marker for both SZ and Alzheimer's disease in Russians [10, 11], but not in Central Asian Kazakh population [18]. Other CSMD1 SNPs were also reported to be associated with cognitive and memory functions in healthy Norwegians [17], with cognitive decline in Alzheimer's disease in patients of European descent [19], as well as with clinical outcomes of SZ in Japanese [20].

Between-population variability in CSMD1 effects on neurocognitive phenotypes may reflect population-specific composition of genetic risk factors and/or population-specific LD patterns of associated markers with unknown functional variant(s) within CSMD1 or in adjacent genetic loci. But, nevertheless, growing amount of data on CSMD1 genetic association with various neuropsychological traits clearly indicates that structural variability at this part of the genome is likely a part of the common neurological mechanism, underlying various diseases and normal variability in neurocognitive traits. Genetic overlapping over these traits may be mediated by cognitive endophenotypes, such as variability in normal cognitive performance due to normal functional differences in brain structure and functions. This point finds support in several functional studies, demonstrating that that SZ risk variants show greater effects at the level of imaging based metrics of brain structure and function than at the level of behavior [21]. Particularly, CSMD1 rs10503253 “A” allele was associated with comparatively reduced cortical activation in the middle occipital gyrus and cuneus during performance of a spatial working memory task [22].

Along with population- and genetic background-related effects, age-related differences also may play a substantial role in the variability in genetic patterns of neurocognitive traits. Association of CSMD1 variants with Alzheimer's disease probably suggests that some part of genetic susceptibility at this locus may appear in the older age, compared to SZ susceptibility variants, but may be expressed in earlier preclinical stages as markers of cognitive and memory performance. However currently we cannot delineate possible molecular mechanisms underlying the age-related difference in CSMD1 effects on neurocognitive functions. The cohort tested in the present study is, to the best of our knowledge, the oldest sample ever used for CSMD1 genetic associations with neurocognitive phenotypes. We observed the association with MoCA performance for the genetic variant previously found to be associated with cognitive functions in younger people [9], as well as with late-onset Alzheimer's disease in our previous work [10]. Thus the findings reported in the current paper support the growing amount of evidence for significant role of CSMD1 in normal and pathological cognitive phenotypes.

## 5. Conclusions

Our study suggests that genetic variants in CSMD1 gene are likely a part of genetic component of cognitive performance in the elderly.

## Figures and Tables

**Table 1:** Demographic and health characteristics of the sample.

Characteristics	Mean ± SD or % where indicated
Age, years	70.9 ± 5.7
Gender, female	74%
Education	
Years of education	13.3 ± 3.1
Min education, years	4
Max education, years	20
Maximum achieved level of education	
Less than high school graduate	11%
High school graduate	8%
Some college or associate's degree	32%
Bachelor's degree	42%
Master's or higher professional degree	
Doctoral degree	7%
Memory testing history	7%
Clinical reasons	3%
Research participant	3%
Health characteristics	
Coronary artery disease	44%
Atrial fibrillation	24%
Stroke	7%
Congestive heart failure	17%
High blood pressure	81%
Obesity	26%
Diabetes, type 2	18%
Smoking	
Smoking during last 30 days	9%
Smoking more than 100 cigarettes over life	20%

**Table 2:** Allele and haplotype frequency of two CSMD1 genetic variants in the total sample and in the lower (Q1) and upper (Q4) quartiles of the MoCA distribution.

Allele, haplotype	Total sample, *N* = 708	Lower quartile (MoCA ≤ 20), *N* = 188	Upper quartile (MoCA ≥ 25), *N* = 193	Q1 versus Q4, *p*
rs1050325				
C	0.733	0.741	0.749	0.799
A	0.267	0.259	0.251	

rs2616984				
A	0.726	0.754	0.674	0.014
G	0.274	0.246	0.326	

rs1050325/rs2616984 haplotypes				
CA	0.524	0.557	0.497	0.101
CG	0.209	0.184	0.251	0.024
AA	0.201	0.197	0.176	0.455
AG	0.065	0.062	0.075	0.478

**Table 3:** Odds ratio values for lower MoCA score for alleles, genotypes, and haplotypes of CSMD1 genetic variants in comparison of lower (Q1) and upper (Q4) quartiles of MoCA score distribution.

Allele, genotype, haplotype	OR	95% CI	Chi-square	*p*
rs10503253				
А	1,04	0.74–1,46	0.06	0,799
C	0.96	0.68–1.35	0.06	0.799
АA	0.95	0.42–2.16	0.01	0.905
CА	1.13	0.73–1.76	0.34	0.562
CC	0.90	0.58–1.37	0.29	0.592

rs2616984				
**A**	**1,49**	**1,07**–**2,07**	**6,01**	**0,014**
**G**	**0.67**	**0.48**–**0.94**	**6.01**	**0.0142**
**AA**	**1.70**	**1.11**–**2.61**	**6.64**	**0.0099**
**AG**	**0.65**	**0.42**–**1.00**	**4.25**	**0.039**
GG	0.70	0.32–1.51	0.96	0.326

rs10503253/rs2616984 haplotypes				
CA	1,27	0,94–1,70	2,63	0,105
CG	0,67	0,47–0,97	4,97	0,026
AA	1,15	0,79–1,69	0,59	0,443
AG	0,81	0,44–1,47	0,55	0,457

Significant values are in bold.

**Table 4:** One-way ANOVA analysis of MoCA scores among genotypes of genetic variants in CSMD1 gene.

Genotype	*N*	MoCA mean	MoCA std. deviation
rs1050325, *F* = 0.105, *p* = 0.899			
CA	247	22.08502	3.747744
AA	57	22.35088	3.319269
CC	370	22.14595	4.159463
All	674	22.14095	3.942305

rs2616984, *F* = 2.814, *p* = 0.060			
AA	361	21.81717	3.966503
AG	255	22.57255	3.928699
GG	57	22.31579	3.728069
All	673	22.14562	3.943373

rs1050325/rs2616984, *F* = 0.860, *p* = 0.549			
AA/AA	37	22.43243	3.484414
CA/AA	126	21.70635	3.655281
CC/AA	198	21.77273	4.239512
AA/AG	16	22.25000	3.193744
CA/AG	100	22.55000	3.957795
CC/AG	139	22.62590	4.005926
AA/GG	3	23.00000	2.645751
CA/GG	21	22.14286	3.119066
CC/GG	33	22.36364	4.211726
All	673	22.14562	3.943373
